# Pharmacokinetics and Safety of Lurbinectedin Administrated with Itraconazole in Cancer Patients: A Drug–Drug Interaction Study

**DOI:** 10.3390/md22040178

**Published:** 2024-04-16

**Authors:** Irene Moreno, Tatiana Hernández, Emiliano Calvo, Salvador Fudio, Carmen Kahatt, Sara Martínez, Jorge Luis Iglesias, Román Octavio Calafati, Laura Pérez-Ramos, Lola Montilla, Ali Zeaiter, Rubin Lubomirov

**Affiliations:** 1 START Madrid—Centro Integral Oncológico Clara Campal (CIOCC), Hospital Universitario HM Sanchinarro, 28050 Madrid, Spain; 2 START Madrid—FJD, Hospital Universitario Fundación Jiménez Díaz, 28040 Madrid, Spain; 3 PharmaMar S.A., 28770 Colmenar Viejo, Spainsmgonzalez@pharmamar.com (S.M.);

**Keywords:** drug interactions, cytochrome P450, pharmacokinetics, cancer

## Abstract

This open-label, two-part, phase Ib drug–drug interaction study investigated whether the pharmacokinetic (PK) and safety profiles of lurbinectedin (LRB), a marine-derived drug, are affected by co-administration of itraconazole (ITZ), a strong CYP3A4 inhibitor, in adult patients with advanced solid tumors. In Part A, three patients were sequentially assigned to Sequence 1 (LRB 0.8 mg/m^2^, 1-h intravenous [IV] + ITZ 200 mg/day oral in Cycle 1 [C1] and LRB alone 3.2 mg/m^2^, 1 h, IV in Cycle 2 [C2]). In Part B, 11 patients were randomized (1:1) to receive either Sequence 1 (LRB at 0.9 mg/m^2^ + ITZ in C1 and LRB alone in C2) or Sequence 2 (LRB alone in C1 and LRB + ITZ in C2). Eleven patients were evaluable for PK analysis: three in Part A and eight in Part B (four per sequence). The systemic total exposure of LRB increased with ITZ co-administration: 15% for C_max_, area under the curve (AUC) 2.4-fold for AUC_0–t_ and 2.7-fold for AUC_0–∞_. Co-administration with ITZ produced statistically significant modifications in the unbound plasma LRB PK parameters. The LRB safety profile was consistent with the toxicities described in previous studies. Co-administration with multiple doses of ITZ significantly altered LRB systemic exposure. Hence, to avoid LRB overexposure when co-administered with strong CYP3A4 inhibitors, an LRB dose reduction proportional to CL reduction should be applied.

## 1. Introduction

**Figure 1:**
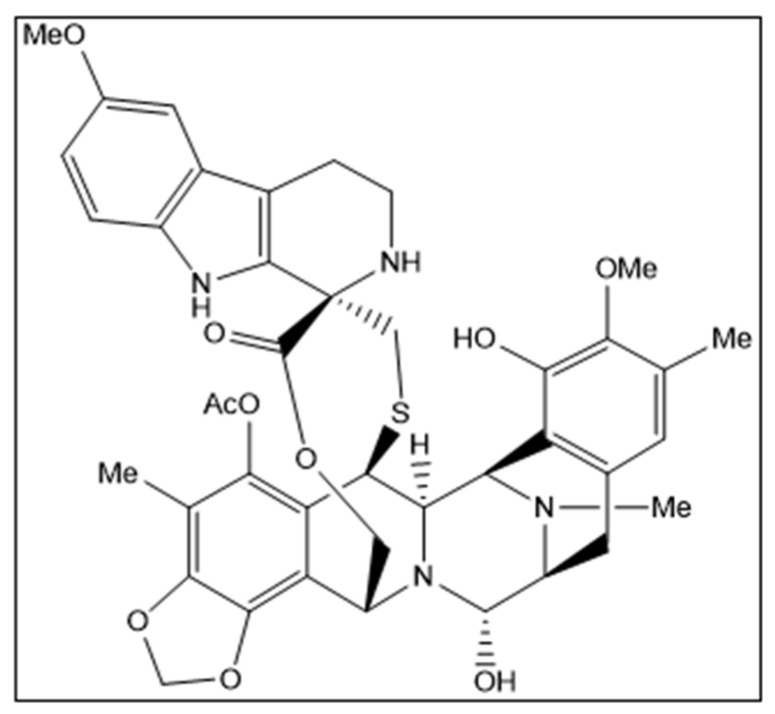
Structural formula of lurbinectedin. Chirality/Stereochemistry: lurbinectedin is a single stereoisomer with the (1′*R*, 6*R*, 6a*R*, 7*R*, 13*S*, 14*S*, 16*R*) configuration.

Lurbinectedin (Zepzelca^TM^) is a novel marine-derived chemical entity structurally related to trabectedin consisting of a monobridged pentacyclic skeleton composed of two fused tetrahydroisoquinoline rings linked to a 10-membered lactone bridge through a benzylic sulfide linkage with an additional tetrahydro-β-carboline moiety (Figure 1). The original marine source, *Ecteinascidia turbinata*, is a colonial ascidian (tunicate) species with a transparent, orange or whitish-colored tunic. Ascidians, or sea squirts, are small, bottom-dwelling soft-bodied marine animals that form colonies comprising many individuals, called zooids [1,2,3].

Lurbinectedin inhibits oncogenic transcription primarily through binding to the exocyclic amino group of guanine-rich DNA sequences around promoters of protein-coding genes, thereby altering the 3D DNA structure and evicting oncogenic transcription factors from their binding sites [4,5,6]. Lurbinectedin adducts can also stop transcribing (phosphorylated) RNA polymerase II, decreasing mRNA synthesis and inducing the ubiquitination and degradation of RNA polymerase II inhibition [7]. Lurbinectedin may also trick the nucleotide excision repair system, favoring the production of DNA double-strand breaks and triggering apoptotic cell death [8]. Lurbinectedin was first approved in June 2020 by the U.S. Food and Drug Administration (FDA) [9,10] and later in several other countries for the treatment of adult patients with metastatic small cell lung cancer (SCLC) with disease progression on or after platinum-based chemotherapy. The FDA granted accelerated approval to lurbinectedin based on the results of a Basket phase II study (ClinicalTrials.gov: NCT02454972) [11]. Lurbinectedin has shown promising results not only in SCLC but also in other solid tumors such as relapsed Ewing sarcoma, endometrial cancer, neuroendocrine tumors, and germline BRCA1/2 metastatic breast cancer [12,13,14,15]. Lurbinectedin is recommended in the United States National Comprehensive Cancer Network (NCCN) guidelines [16] and the European Society of Medical Oncology (ESMO) [17] as a treatment option for relapsed SCLC. Currently, a randomized phase III confirmatory study (LAGOON; ClinicalTrials.gov: NCT05153239) is evaluating lurbinectedin alone or combined with irinotecan versus standard-of-care therapy in second-line SCLC [18].

Lurbinectedin is highly protein-bound. Based on in vitro studies, metabolism of lurbinectedin by cytochrome P450 (CYP) 3A is the major clearance (CL) mechanism. A population pharmacokinetic (PopPK) model [19] was developed with data from 443 cancer patients treated in six phase I and three phase II trials with 1 h intravenous (IV) infusion of lurbinectedin as a single agent or combined with other agents. The population estimate for total plasma CL was 11.2 L/h, corresponding to a blood CL of ~17 L/h, thus reflecting a low extraction ratio of 0.19. The population estimate of apparent volume at steady state was 438 L. High α-1-acid glycoprotein and C-reactive protein and low albumin reduced CL by 28%, 20%, and 20%, respectively. Co-administration of cytochrome CYP3A inhibitors reduced CL by 30% [19].

There is a consensus that itraconazole is the most appropriate replacement for ketoconazole in clinical drug–drug interaction studies, given its strong CYP3A4 inhibition, extensive clinical experience in drug–drug interaction studies, low QTc prolongation in the electrocardiogram potential, and well-known safety profile [20,21]. The itraconazole safety profile is characterized by mild and transient liver enzymes elevations, reported in <5% of patients with chronic dosing, and with an estimated frequency of 1:500,000 for clinically relevant hepatotoxicity. Short-term dosing with itraconazole (i.e., ≤14 days), as in drug–drug interaction studies, is associated with a very low risk of liver injury compared to chronic treatment. The risk of QTc prolongation in the electrocardiogram with itraconazole administration, especially at a limited dose (i.e., ≤200 mg once a day, qd), is low based on in vitro data and clinical experience [20]. The only safety concern associated with itraconazole is the risk of decreased cardiac contractility [22].

Cytochrome P450 3A (CYP3A) enzymes are often significant contributors to the CL of drugs; therefore, strong inhibitors that are selective for these enzymes are used in clinical drug–drug interaction studies [21]. As lurbinectedin is primarily metabolized in the liver by CYP3A4 [23], a strong inhibitor of this enzyme such as itraconazole may affect the CL rates and plasma exposures of lurbinectedin. Consequently, in this drug–drug interaction study, the proposed itraconazole regimen (i.e., 200 mg once-daily during 12 days, 8 days after the start of lurbinectedin infusion) was expected to induce sustained inhibition of CYP3A4 activity and ABCB1 (P-gp) (a drug resistance protein) transport over the entire lurbinectedin pharmacokinetic (PK) profile [20,21]. Previously, a lurbinectedin dose of 1.3 mg/m^2^ administered over 1 h without concomitant administration of CYP3A4 inhibitors produced concentrations in plasma that were measurable for at least 160 h after starting the infusion [24]. A lurbinectedin dose of 0.8 mg/m^2^ co-administered with itraconazole was expected to produce measurable concentrations in samples collected for at least the initial 160 h. The plasma exposure values of lurbinectedin at 0.8 mg/m^2^ were not expected to exceed those observed following administration of lurbinectedin alone at 3.2 mg/m^2^ given over 1 h, since the concomitant administration of aprepitant (a moderate CYP3A4 inhibitor) decreased the mean plasma CL of lurbinectedin by only 30%.

This manuscript presents the results of a dedicated drug–drug interaction study designed to evaluate the PK and safety of lurbinectedin co-administered with a strong CYP3A4 inhibitor (itraconazole) in patients with advanced malignancies. These findings have been used to inform the product labeling of lurbinectedin, providing the appropriate dose reduction required when co-administered with a strong CYP3A4 inhibitor.

## 2. Results

### 2.1. Patient Disposition and Baseline Characteristics

**Figure 2:**
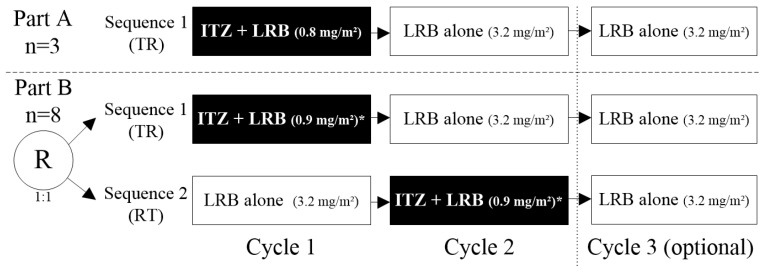
Study design. Note: eleven evaluable patients (three in Part A and eight in Part B) were planned to be enrolled in the study. Finally, fourteen patients were included and treated: three in Part A/Sequence 1 [TR] and eleven in Part B, of them five in Sequence 1 [TR] and six in Sequence 2 [RT]. * In Part B, the dose of lurbinectedin when co-administered with itraconazole was increased to 0.9 mg/m^2^, based on exposure and safety experience in Part A. ITZ, itraconazole; LRB, lurbinectedin; R; randomized; RT, Reference–Test; TR, Test–Reference; PK, pharmacokinetic.

Fourteen patients were included and treated: three patients in Part A in Sequence 1 (TR: Test [itraconazole + lurbinectedin in Cycle 1]—Reference [lurbinectedin alone]), and eleven patients in Part B (five in Sequence 1 [TR] and six in Sequence 2 [RT: Reference [lurbinectedin alone]—Test [itraconazole + lurbinectedin in Cycle 2]). Cycle 3 of lurbinectedin alone was optional in both sequences (Figure 2). In Part A, all patients were sequentially assigned to Sequence 1, while in Part B, patients were randomly assigned (1:1) to Sequence 1 or Sequence 2. Eligibility criteria are described in Section 4.1.

**Figure 3:**
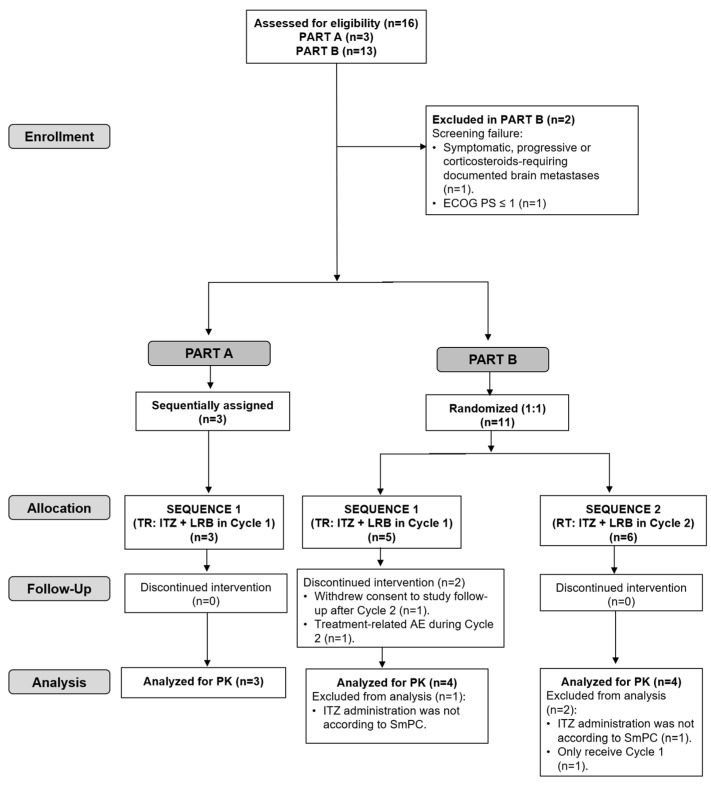
CONSORT flow diagram for the trial. ECOG PS, Eastern Cooperative Oncology Group performance status; ITZ, itraconazole; LRB, lurbinectedin; RT, Reference–Test (ITZ + LRB in Cycle 2); TR, Test–Reference (ITZ + LRB in Cycle 1); PK, pharmacokinetics; SmPC, Summary of Product Characteristics.

Five of the fourteen included patients (36%) discontinued treatment due to progressive disease (two in Part A and three in Sequence 2 of Part B). In Sequence 1 of Part B, one patient discontinued treatment due to a treatment-related adverse event (AE) during Cycle 2 and one patient refused to continue treatment (see the CONSORT flow diagram in Figure 3).

The main patient characteristics at baseline by study part (Part A and Part B) and sequence (Sequence 1 [TR] and Sequence 2 [RT]) are summarized in the Supplementary Material Table S1. Nine of the fourteen included patients (64%) were female. The median age was 63 years (range, 49–72 years); 69 years (range, 49–72 years) in Part A and 64 years (range, 56–70 years) in Part B. The performance status score (ECOG PS) of all patients was 0–1, with most of them (71%) having score 0. The most common primary tumors were ovarian carcinoma (*n* = 4; 29%), lung (*n* = 3; 21%), and endometrial carcinoma (*n* = 2; 14%). The median number of disease sites at baseline was 3 (range, 1–5 sites), with 50% of patients having ≥3 sites. The most common disease sites were lung and lymph nodes (*n* = 9; 64% of patients each). The median time from disease diagnosis to first infusion was 3.4 years (range, 1.0–13.8 years).

### 2.2. Pharmacokinetics

Eleven patients who received at least one lurbinectedin dose completed PK sampling in Cycles 1 and 2 and had sufficient and interpretable PK assessments, which were included in the PK analysis set (Part A/Sequence 1, *n* = 3; Part B, *n* = 8, with 4 in each of Sequences 1 and 2).

Of the fourteen treated patients, three patients in Part B (one in Sequence 1 and two in Sequence 2) were excluded from the PK analysis population: two patients (one in each Sequence 1 and 2) because itraconazole was not self-administered according to the product label recommendations in case of co-administration with gastric acid secretion suppressants and one patient in Sequence 2 because he did not receive Cycle 2 (Figure 3).

#### 2.2.1. Total and Unbound Plasma Lurbinectedin Pharmacokinetics

**Figure 4:**
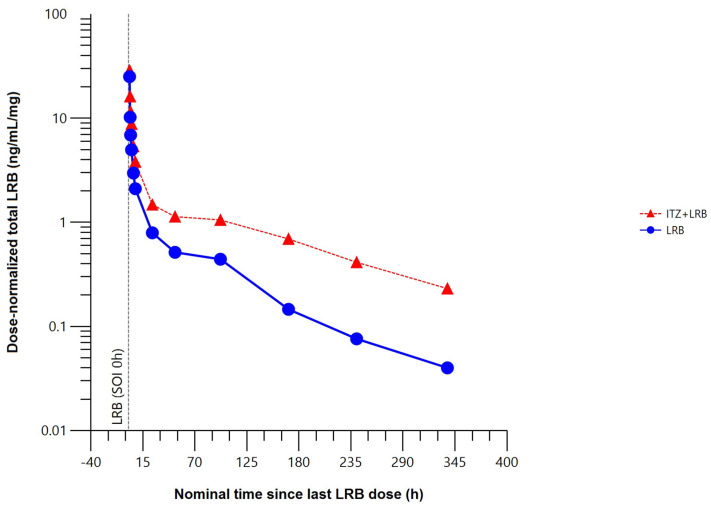
Mean dose–normalized total plasma concentration–time profile of lurbinectedin with (*n* = 8) or without (*n* = 8) itraconazole (pharmacokinetic data analysis set). h, hour; ITZ, itraconazole; LRB, lurbinectedin; SOI, start of infusion.

The mean total plasma concentration–time profile of lurbinectedin was higher when co-administered with itraconazole (Figure 4).

Compared to lurbinectedin alone, co-administration with itraconazole affected the extent of exposure, increasing the maximum plasma concentration (C_max_) of lurbinectedin by 15%, and the concentration–time curve (AUC) from time 0 to the time of the last quantifiable concentration (AUC_0–t_) by 2.4-fold and from time 0 to infinity (AUC_0–∞_) by 2.7-fold. Co-administration with multiple oral doses of itraconazole reduced total plasma lurbinectedin CL by 63% and prolonged terminal half-life (t_1/2_) by 2.2-fold. These changes in lurbinectedin CL, terminal elimination half–life (t_1/2_), and systemic exposure were statistically significant at the 90% confidence interval (CI) level (Table 1).

**Figure 5:**
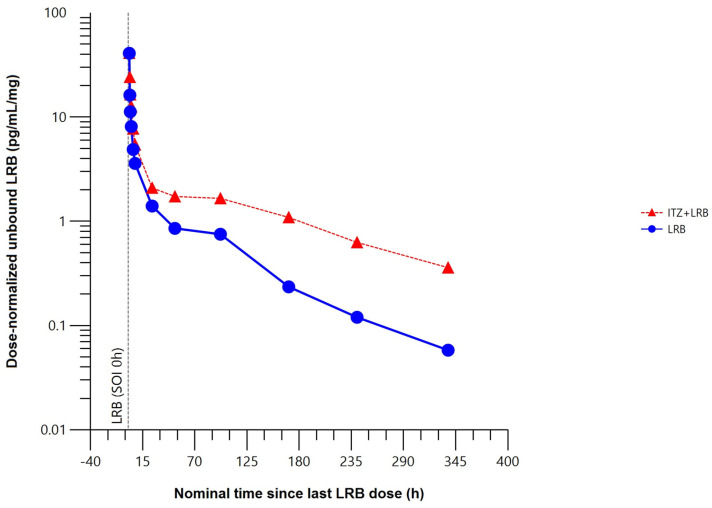
Mean dose–normalized unbound plasma concentration–time profile of lurbinectedin with (*n* = 5) or without (*n* = 8) itraconazole (pharmacokinetic data analysis set). h, hour; ITZ, itraconazole; LRB, lurbinectedin; SOI, start of infusion.

The mean unbound plasma concentration–time profile of lurbinectedin was higher when co-administered with itraconazole (Figure 5).

Compared to lurbinectedin alone, co-administration with itraconazole produced statistically significant changes in the unbound plasma lurbinectedin PK parameters to a similar extent to those in total plasma lurbinectedin PK. Co-administration with itraconazole significantly increased systemic exposure of unbound lurbinectedin (i.e., 2.2-fold in AUC_0–t_ and 2.4-fold in AUC_0–∞_). Unbound lurbinectedin CL decreased by 58% and t_1/2_ was 2-fold longer. Changes in unbound lurbinectedin CL, t_1/2_, and systemic exposure were statistically significant at the 90% CI level (Table 2).

#### 2.2.2. Lurbinectedin Metabolites (M1 and M4) Plasma Pharmacokinetics

Co-administration with itraconazole almost completely inhibited the conversion of lurbinectedin to metabolite M1 (1′,3′–dihydroxy–lurbinectedin) and reduced by 69% the conversion of lurbinectedin to metabolite M4 (N–desmethyl–lurbinectedin, PM030047) compared with lurbinectedin administered alone (Table 3).

### 2.3. Safety

Safety analyses included all 14 patients who received at least one dose of lurbinectedin (Part A, *n* = 3; Part B, *n* = 11). Thirteen of the fourteen treated patients (three in Part A and ten in Part B) received itraconazole plus lurbinectedin in Cycles 1 or 2, and all fourteen treated patients (three in Part A and 11 in Part B) received lurbinectedin alone in Cycles 1, 2, or 3.

A total of 36 cycles were administered (nine in Part A/Sequence 1, and twenty-seven in Part B/Sequences 1 and 2). In Part B, 13 cycles were given in Sequence 1 (TR: itraconazole + lurbinectedin [ITZ + LRB] in Cycle 1 and LRB alone in Cycle 2) and 14 were given in Sequence 2 (RT: LRB alone in Cycle 1 and ITZ + LRB in Cycle 2), with a median number of 3 cycles (range, 1–3 cycles) per patient and a median relative dose intensity of 98.3% (range, 77.9–100.1%) in all treated patients.

One patient (33%) in Part A, and six patients in Part B (n = 3, 60% in Sequence 1 and *n* = 3, 50% in Sequence 2) continued treatment under compassionate use after completion of the optional Cycle 3.

Treatment-related AEs (>10% of patients or grade ≥ 3) and laboratory abnormalities (regardless of relationship) by study part (regardless of sequence) and according to the worst grade per treatment are shown in Table 4. No grade ≥ 3 AEs related to treatment (or with unknown relationship) were observed while patients were in treatment with the combination of ITZ + LRB in Part A/Sequence 1 or Part B (Sequences 1 and 2). Only one patient discontinued treatment due to a treatment-related serious AE (grade 3 rhabdomyolysis related to both study drugs) that occurred in Cycle 2 of Part B (Sequence 1) with lurbinectedin alone. Two patients had a lurbinectedin dose reduction due to AEs during treatment with lurbinectedin alone: one patient in Part A (Sequence 1) required a dose reduction to 2.6 mg/m^2^ in Cycle 3 due to lurbinectedin-related grade 3 alanine aminotransferase increasing in Cycle 2 and one patient in Part B (Sequence 2) had a dose reduction to 0.7 mg/m^2^ in combination with itraconazole in Cycle 2 due to lurbinectedin-related grade 4 neutropenia during Cycle 1. No treatment-related deaths occurred in this study. All treatment-emergent adverse events (TEAEs) of any grade regardless of relationship by study part are provided in the Supplementary Material (Table S2).

## 3. Discussion

Itraconazole is a strong CYP3A4 and P-gp inhibitor that has been widely used in drug–drug interaction studies with anticancer drugs [25,26,27,28]. Regulatory agencies currently recommend the use of this strong CYP3A4 inhibitor in the design of clinical drug–drug interaction studies [21]. Lurbinectedin (a second-generation trabectedin analog) is primarily metabolized by the CYP3A4 isoenzyme; therefore, potent CYP3A4 inhibitors such as itraconazole are expected to affect the PK profile of lurbinectedin when given concomitantly. In this phase Ib drug–drug interaction study of lurbinectedin and itraconazole in patients with advanced solid tumors, the use of a crossover design allowed us to reduce treatment bias by using the subjects themselves (intra-subject comparison) as a control group.

The starting dose of lurbinectedin given in combination with itraconazole for the first three patients of the first part (Part A) of the study was 0.8 mg/m^2^; however, in the second part (Part B) of the study, this dose was increased to 0.9 mg/m^2^ based on exposure data and the favorable safety profile observed in Part A. From a safety point of view, no clinically relevant differences were observed between these two doses (0.8 and 0.9 mg/m^2^).

Co-administration of multiple oral doses of itraconazole with a single lurbinectedin dose had an effect on exposure, increasing total lurbinectedin C_max_ by 15%, AUC_0–t_ by approximately 2.4-fold, and AUC_0–∞_ by approximately 2.7-fold, reducing CL by 63% and prolonging t_1/2_ by 2.2-fold. Similarly, co-administration with itraconazole increased systemic exposure of unbound lurbinectedin by approximately 2.2-fold for AUC_0–t_ and by 2.4-fold for AUC_0–∞_, reduced CL by 58%, and prolonged t_1/2_ by 2-fold. The magnitude of the observed changes showed that co-administration of itraconazole had a clinically relevant effect on lurbinectedin PK in patients with advanced solid tumors. Such an effect of itraconazole is a consequence of a sustained inhibition of the activity of CYP3A4 and ABCB1 (P-gp) (a drug resistance protein) consistent with the in vitro characterization of the metabolic and elimination profile of lurbinectedin.

The design of this trial was adequate for characterizing the PK profile of total and unbound lurbinectedin administered alone and in combination with itraconazole. The results indicate that lurbinectedin dosage adjustments are required when strong CYP3A inhibitors are co-administered. Therefore, to avoid lurbinectedin overexposure, a dose modification of lurbinectedin (i.e., a dose reduction proportional to CL reduction) is necessary when administered with concomitant medications that strongly inhibit CYP3A. Consequently, this information was added to the Prescribing Information (Label) for Zepzelca^TM^ (lurbinectedin) [29].

Lurbinectedin 0.9 mg/m^2^ administered as a 1 h IV infusion on Day 1 every three weeks (q3wk) with itraconazole was well tolerated. The safety profile of lurbinectedin alone at 3.2 mg/m^2^ as a 1 h IV infusion q3wk was consistent with that reported for this dose and schedule in previous phase II and III trials in patients with advanced cancer, which consisted of neutropenia, nausea, vomiting, and fatigue as the most treatment-related AEs [30].

As expected, the safety profile of lurbinectedin administered at the adjusted dose of 0.9 mg/m^2^ when co-administered with itraconazole was not worse than that of lurbinectedin administered alone at the recommended dose of 3.2 mg/m^2^.

## 4. Materials and Methods

This clinical trial was conducted at two centers in Spain in accordance with the ethical principles originating in the Declaration of Helsinki and in compliance with ICH Good Clinical Practice guidelines and applicable regulatory requirements and in compliance with the protocol. The study protocol was approved by the Spanish Agency of Medicines and Medical Devices (AEMPS) (protocol code: 2020-001332-96 approved on 21 August 2020) and the Independent Ethics Committee of HM Hospitals/Spain (20.05.1632-GHM approved on 19 August 2019), and it was registered with the EU Clinical Trials Register EudraCT (2020–001332-96) and ClinicalTrials.gov Trials Register (NCT05063318). Signed written informed consent was obtained for each patient before study-specific procedures.

### 4.1. Study Population

Eligible patients were men and women aged ≥ 18 years, with pathologically confirmed advanced solid tumors for which no suitable effective therapy existed; life expectancy > 3 months; who had recovered from previous toxicities to grade ≤ 1 (excluding alopecia and grade 1/2 asthenia or fatigue); with ECOG PS score ≤ 1; and with adequate organ function. Women were postmenopausal, surgically sterile, abstinent or practicing a highly effective method of birth control (including breast feeding) throughout the study, and for six months thereafter. Men used an adequate contraception method (e.g., vasectomy, double barrier, partner using effective contraception). Major exclusion criteria included the prior use of strong or moderate inhibitors or inducers of CYP3A4 activity within three weeks prior to Day 1 of Cycle 1 and use of CYP3A4 substrates such as HMG-CoA reductase inhibitors atorvastatin, lovastatin, and simvastatin, for which concomitant administration with strong CYP3A4 inhibitor was contraindicated; and treatment with any investigational drug within 30 days before of Day 1 of Cycle 1. Patients with central nervous system metastasis, cirrhosis, alcohol-induced steatosis, chronic active hepatitis infection, significant cardiovascular conditions, and medical conditions such as obstructive cholestatic liver disease (suitable for stenting procedure) or biliary sepsis in the past two months, active COVID-19 disease, or psychiatric disorders were also excluded.

### 4.2. Study Design

This was an open-label, two-part, crossover, phase Ib drug–drug interaction study conducted in adult patients with advanced solid tumors. The study was designed to evaluate the drug–drug interaction of lurbinectedin (a marine-derived drug) and itraconazole (a strong CYP3A4 inhibitor) and included the following phases: (a) screening phase (within 14 days prior to any study procedure); (b) treatment phase; and (c) follow-up phase after the last dose of lurbinectedin.

The study consisted of two parts. In Part A, three patients were sequentially enrolled to receive Sequence 1 (TR: Test–Reference) consisting of itraconazole (200 mg tablets; once-daily for 12 days) co-administered with lurbinectedin (0.8 mg/m^2^, 1 h, IV infusion q3wk) (Cycle 1), followed by two consecutive cycles (Cycles 2 and 3) of lurbinectedin alone (3.2 mg/m^2^, 1 h, IV infusion q3wk), with the third cycle being optional. In Part B, patients were randomized (in a ratio 1:1, using sequence a block randomization implemented with Medidata Rave Randomization and Trial Supply Management [RTSM]) to receive either Sequence 1 (TR: Test–Reference), but with lurbinectedin 0.9 mg/m^2^, 1 h, IV when co-administered with itraconazole, or Sequence 2 (RT: Reference–Test) of lurbinectedin alone (Cycle 1), followed by itraconazole and lurbinectedin co-administration (Cycle 2), with lurbinectedin alone (optional Cycle 3) (see Figure 2 above).

Part A was performed to determine the dose of lurbinectedin that would be safe to be co-administered with itraconazole; if necessary, this dose could be adjusted in Part B. Based on exposure data and the safety experience observed in Part A, the dose of lurbinectedin when co-administered with itraconazole was increased to 0.9 mg/m^2^ in Part B. The dose of lurbinectedin alone in Part A and Part B was 3.2 mg/m^2^ q3wk for all patients [31]; this dose has been used in studies of single-agent lurbinectedin conducted with cancer patients in the US and Europe [11,24,32] and is the approved dose for relapsed SCLC patients [9].

**Figure 6:**
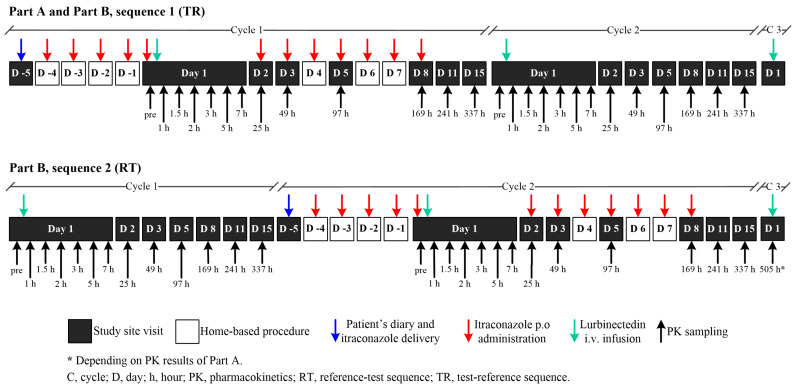
Schematic diagram of trial design. Part A: Sequence 1; Part B: Sequence 1 or Sequence 2 (1:1 ratio). In Part A, three patients were sequentially enrolled to receive Sequence 1 (TR) of ITZ with LRB (Cycle 1) followed by two consecutive cycles (Cycle 2 and the optional Cycle 3) of LRB alone. In Part B, patients were randomized (1:1) to receive either Sequence 1 (TR, as described in Part A) or Sequence 2 (RT) of LRB alone (Cycle 1), followed by ITZ and LRB (Cycle 2) and LRB alone (optional Cycle 3). ITZ, itraconazole; LRB, lurbinectedin; RT, Reference–Test; TR, Test–Reference.

The itraconazole dose (200 mg) was selected based on the published literature for the design of clinical drug–drug interaction studies with itraconazole [19]. In the co-administration cycles, itraconazole was administered as 200 mg (2 × 100 mg capsules) once-daily doses in the morning after breakfast for 12 consecutive days, self-administered at home starting from Day 4 (i.e., four days before lurbinectedin infusion) to Day 8 (i.e., seven days after lurbinectedin infusion), following recommendations from the Summary of Product Characteristics. On Day 1 (day of lurbinectedin infusion), itraconazole was given immediately prior to the start of the lurbinectedin infusion. In fact, itraconazole had to be administered after collecting the first itraconazole PK sample and before the start of lurbinectedin infusion (−15 min to −1 min). In case of lurbinectedin delay (≤2 days), itraconazole could be administered for a maximum of 14 days (Figure 6). In Sequence 1 (TR), itraconazole for self-administration and the patient’s diary were given to the patient on Day −5 with a −2/+1 daytime window (i.e., from Day −7 to Day −4). In Sequence 2 (RT), itraconazole and the patient’s diary were given to the patient on Day −5 of Cycle 2 with a −2/+1 daytime window (i.e., from Day 15 of Cycle 1 to Day −4 of Cycle 2).

### 4.3. Pharmacokinetic Assessments

The impact of itraconazole on the lurbinectedin PK profile compared with that of lurbinectedin alone was evaluated using the primary study PK endpoints: C_max_ and AUC_0–∞_ of total plasma lurbinectedin. Other secondary PK endpoints included AUC_0–t_, total plasma CL, t_1/2_, and the volume of distribution at steady state (V_ss_). The following unbound parameters were also estimated: AUC_u,0–∞_, AUC_u,0–t_, C_u,max_, CL_u_, V_ss,u_, and t_1/2,u._ Plasma PK parameters were calculated with a standard non–compartmental analysis approach using Phoenix^®^ WinNonlin^®^ v6.4 (Certara USA, Inc., Princeton, NJ, USA).

Serial peripheral venous blood samples for the lurbinectedin PK analysis (and its metabolites M1 and M4) were collected in Cycle 1 and Cycle 2 (Sequences 1 and 2), with a schedule of thirteen samples in all patients as follows: at pre-dose, 5 min prior to the end of the lurbinectedin infusion, and at 0.5, 1, 2, 4, 6, 24, 48, 96, 168, 241, and 337 h post-dose on Days 1, 2, 3, 5, 8, 11, and 15. Only in Part B (Sequence 2) was a blood sampling test at 505 h post-lurbinectedin dosing to be collected before the lurbinectedin infusion started on Day 1 of Cycle 3, if detectable lurbinectedin concentrations were present in the pre-infusion sample in Cycle 2 of Part A. Blood samples for the PK analysis of itraconazole (and its active metabolite, hydroxy-itraconazole) were collected in cycles with itraconazole co-administration (i.e., Cycle 1 of Part A and B [Sequence 1] and Cycle 2 of Part B [Sequence 2]) at pre-dose and at 1, 2, 4, 24, and 168 h post-itraconazole dosing on Days 1, 2, and 8.

#### Bioanalytical Procedures

A validated Ultra Performance Liquid Chromatography coupled to the tandem Mass Spectrometry (UPLC-MS/MS) method (Dynakin, S.L., Derio, Spain) was used to determine the total plasma concentrations of lurbinectedin and its metabolites M1 and M4 [33]. A validated UPLC-MS/MS method was used to determine by rapid equilibrium dialysis and quantification the lurbinectedin unbound fraction in plasma [33]. The lower limits of quantification of total lurbinectedin, unbound lurbinectedin, and M1 and M4 metabolites were 0.1 ng/mL, 0.02%, 0.5 ng/mL, and 0.1 ng/mL, respectively. Itraconazole and its metabolite, hydroxy-itraconazole plasma concentrations, aiming to assure adequate treatment compliance, was determined using Liquid Chromatography coupled to the tandem Mass Spectrometry (LC-MS/MS) method (Q Squared Solutions, Ithaca, NY, USA). The lower limit of quantification for itraconazole and its metabolite was 1.0 ng/mL.

### 4.4. Safety Assessment

Safety evaluations included assessments of AEs, clinical laboratory tests, deaths, vital signs measurements, and physical examinations. TEAEs were defined as any AE that worsened in severity from baseline or having their onset between the first dose of the study drug and 31 days (±10 days) after the last dose, death, or date of further therapy. AEs were coded based on the Medical Dictionary for Regulatory Activities (MedDRA) v. 23.0 and graded as per the National Cancer Institute–Common Terminology Criteria for Adverse Events (NCI-CTCAE) v. 5.0.

### 4.5. Statistical Methods

#### 4.5.1. Statistical Analysis

The plasma dose-normalized AUC_0–∞_ of lurbinectedin (AUC_0–t_ was used if AUC_0–∞_ could not be calculated due to insufficient available data) was the primary parameter of interest for the statistical analysis, which compared the log-transformed AUC for lurbinectedin in combination with itraconazole (Test) versus lurbinectedin alone (Reference). A mixed-effects model was fitted to the data with log-transformed AUC as the dependent variable, treatment (Test or Reference), period (cycle), and sequence as fixed effects, and the patient as a random effect. The estimated least-square means and intra-subject variability from the mixed-effects model were used to construct 90% CIs for the difference in means on the log scale between treatments. A large difference (e.g., a two-fold difference in CIs and least-square means) was considered indicative of a clinically relevant effect of itraconazole co-administration on lurbinectedin exposure. For the secondary PK parameters, similar models used for the primary endpoint were fitted to the data with a dose-normalized AUC_(0–t)_ and C_max_, and in Cl, the V_ss_ and t_1/2_ of lurbinectedin or their metabolites to the parent exposure PK parameters ratio, as the dependent variable. In addition, for the plasma protein biding, a similar model was fitted to the data with a dose-normalized AUC_u_ as the dependent variable. Safety results were presented descriptively by the treatment group (Test: ITZ + LRB versus Reference: LRB alone) for each study part (Part A and Part B). SAS^®^ v.9.4 (SAS Institute Inc., Cary, NC, USA) was used for all statistical analysis outputs.

#### 4.5.2. Sample Size Calculation

At least eleven patients (three in Part A and eight in Part B) were expected to complete all study procedures, including the collection of sufficient and interpretable PK assessments. Considering a dropout rate of approximately 30%, a sample size of eight patients was deemed sufficient to determine the impact of itraconazole on the primary endpoints.

The intra-subject CV for the PK parameters of lurbinectedin was estimated to be ≥30%. The half-width of the 90% CI for [(Test: ITZ + LRB)/(Reference: LRB alone)] comparison on the log-scale was extended 0.389 from the observed differences in means, assuming that the intra-subject CV was 40%. This half-width corresponds to a 90% CI in the range of 70% and 147%, assuming the ratio of the means is equal to unity for each PK parameter.

## 5. Conclusions

In summary, co-administration of lurbinectedin with strong CYP3A inhibitors should be avoided, but if required, appropriate lurbinectedin dose reductions should be considered. The findings from this phase Ib study were used for labelling purposes to recommend appropriate dose modifications when patients with advanced solid tumors need to take lurbinectedin concomitantly with strong CYP3A inhibitors.

## Figures and Tables

**Table 1:** Estimated geometric means and ratio with 90% confidence interval between plasma pharmacokinetic parameters of lurbinectedin with and without itraconazole.

PK Parameter (Units)	Treatment ^b^	Geometric Mean (CV%)	Ratio (%) ^c^	90% CI (%) ^d^	Intra-Subject CV (%)
C_max_ (µg/L/mg) ^a^	ITZ + LRB (T)	25.91 (53.15)	115.51	(100.07–133.33) *	14.85
	LRB (R)	22.43 (53.75)			
AUC_0–t_ (µg·h/L/mg) ^a^	ITZ + LRB (T)	244.77 (91.89)	236.73	(177.35–316.00) *	30.40
	LRB (R)	103.4 (78.85)			
AUC_0–∞_ (µg·h/L/mg) ^a^	ITZ + LRB (T)	288.55 (73.9)	272.73	(213.22–348.86) *	25.75
	LRB (R)	105.8 (77.37)			
CL (L/h)	ITZ + LRB (T)	3.47 (73.9)	36.67	(28.66–46.9) *	25.75
	LRB (R)	9.45 (77.37)			
t_1/2_ (h)	ITZ + LRB (T)	101.36 (61)	217.57	(151.81–311.82) *	38.35
	LRB (R)	46.59 (17.12)			
V_ss_ (L)	ITZ + LRB (T)	418.68 (111.45)	99.89	(65.76–151.74)	45.11
	LRB (R)	419.14 (58.58)			

A natural log transformation for AUC and C_max_ was used prior to ANOVA. Geometric means, geometric means ratio, and its 90% CI were back-transformed to the original scale. * Statistically significant at the 90% CI. ^a^ Dose–normalized PK parameter. ^b^ Test, *n* = 8 patients; Reference, *n* = 8 patients. ^c^ Ratio = least-squares geometric mean ratio. Geometric mean ratio was calculated dividing geometric mean of lurbinectedin + itraconazole by geometric mean of lurbinectedin. ^d^ Based on an ANOVA with treatment (T: Test or R: Reference), period (Cycle 1 or 2), and sequence (Sequence 1 [TR] or 2 [RT]) as fixed effects and patient (sequence) as a random effect in the model. ANOVA, analysis of variance; AUC, area under the concentration–time curve; CI, confidence interval; CL, clearance; C_max_, maximum plasma concentration; CV, coefficient of variation; ITZ, itraconazole; LRB, lurbinectedin; n, number of patients with PK parameter included; PK, pharmacokinetic; R, Reference; RT, Reference–Test (ITZ + LRB in Cycle 2); t_1/2_, terminal half-life; T, Test; TR, Test–Reference (ITZ + LRB in Cycle 1); V_ss_, volume of distribution at steady state.

**Table 2:** Estimated geometric means and ratio with 90% confidence interval between unbound plasma pharmacokinetic parameters of lurbinectedin with and without itraconazole.

PK Parameter (Units)	Treatment ^b^	Geometric Mean (CV%)	Ratio (%) ^c^	90% CI (%) ^d^	Intra-Subject CV (%)
C_max_ (pg/mL/mg) ^a^	ITZ + LRB (T)	37.48 (48.84)	97.27	(64.48–146.75) *	30.25
	LRB (R)	40.03 (21.75)			
AUC_0–t_ (pg·h/mL/mg) ^a^	ITZ + LRB (T)	400.28 (63.53)	219.88	(174.82–276.56) *	15.30
	LRB (R)	184.55 (57.58)			
AUC_0–∞_ (pg·h/mL/mg) ^a^	ITZ + LRB (T)	437.95 (63.57)	236.25	(191.53–291.41) *	13.97
	LRB (R)	188.84 (56.92)			
CL (L/h)	ITZ + LRB (T)	2283.37 (63.57)	42.33	(34.32–52.21) *	13.97
	LRB (R)	5295.44 (56.92)			
t_1/2_ (h)	ITZ + LRB (T)	81.81 (21.58)	191.97	(173.75–212.09) *	6.65
	LRB (R)	46.59 (17.12)			
V_ss_ (L)	ITZ + LRB (T)	231,881 (46.39)	98.03	(68.38–140.53)	24.83
	LRB (R)	234,827 (41.9)			

A natural log transformation for AUC and C_max_ was used prior to ANOVA. Geometric means, geometric means ratio, and its 90% CI were back-transformed to the original scale. * Statistically significant at the 90% CI. ^a^ Dose–normalized PK parameter. ^b^ Lurbinectedin unbound fraction cannot be determined in three patients during the ITZ + LRB cycle (Test, *n* = 5 patients; Reference, *n* = 8 patients). ^c^ Ratio = least-squares geometric mean ratio. Geometric mean ratio was calculated dividing geometric mean of lurbinectedin + itraconazole by geometric mean of lurbinectedin. ^d^ Based on an ANOVA with treatment (T: Test or R: Reference), period (Cycle 1 or 2), and sequence (Sequence 1 [TR] or 2 [RT]) as fixed effects and patient (sequence) as a random effect in the model. ANOVA, analysis of variance; AUC, area under the concentration–time curve; CI, confidence interval; CL, clearance; C_max_, maximum plasma concentration; CV, coefficient of variation; ITZ, itraconazole; LRB, lurbinectedin; n, number of patients with PK parameter included; PK, pharmacokinetic; R, Reference; RT, Reference–Test (ITZ + LRB in Cycle 2); t_1/2_, terminal half-life; T, Test; TR, Test–Reference (ITZ + LRB in Cycle 1); V_ss_, volume of distribution at steady state.

**Table 3:** Estimated geometric means and ratio with 90% confidence interval of metabolites M1 and M4/parent ratio in plasma pharmacokinetic parameters between lurbinectedin with and without itraconazole.

Lurbinectedin Metabolites	MPR of PK Parameter (Units) ^a^	Treatment (n)	Geometric Mean (CV%)	Ratio (%) ^d^	90% CI (%) ^e^	Intra-Subject CV (%)
M1 *(1′,3′-dihydroxy-lurbinectedin)*	C_max_ (µg/L/mg)	ITZ + LRB (T) (*n* = 1) ^b^	0.38 (NA)	–	–	–
		LRB (R) (*n* = 8)	0.29 (135.88)			
	AUC_0–t_ (µg·h/L/mg)	ITZ + LRB (T) (*n* = 1) ^b^	0.74 (NA)	–	–	–
		LRB (R) (*n* = 8)	0.48 (360.01)			
M4 (*PM030047*, *N-desmethyl-lurbinectedin)*	C_max_ (µg/L/mg)	ITZ + LRB (T) (*n* = 6) ^c^	0.61 (47.57)	92.47	(55.51–154.05)	44.75
		LRB (R) (*n* = 8)	0.49 (60.3)			
	AUC_0–t_ (µg·h/L/mg)	ITZ + LRB (T) (*n* = 6) ^c^	1.29 (209.58)	31.53	(11.68–85.11)	101.34
		LRB (R) (*n* = 8)	1.45 (135.58)			

A natural log transformation for AUC and C_max_ was used prior to ANOVA. Geometric means, geometric means ratio, and its 90% CI were back-transformed to the original scale. ^a^ Dose-normalized PK parameter. ^b^ Results available only for one patient because all PK samples from the other patients were below the limit of quantification in the ITZ + LRB cycle. Therefore, a formal inferential statistical analysis was not conducted on the log-transformed metabolite/MPR for M1 plasma PK exposure parameters with lurbinectedin alone as reference treatment, because in the co-administration cycles, almost all PK samples of patients, except for one patient, were below the limit of quantification. ^c^ Metabolite M4 plasma samples were below the limit of quantification in two patients in the ITZ + LRB cycle. ^d^ Ratio = least-squares geometric mean ratio. Geometric mean ratio was calculated dividing geometric mean of lurbinectedin + itraconazole by geometric mean of lurbinectedin. ^e^ Based on an ANOVA with treatment (T: Test or R: Reference), period (Cycle 1 or 2), and sequence (Sequence 1 [TR] or 2 [RT]) as fixed effects and patient (sequence) as a random effect in the model. ANOVA, analysis of variance; AUC, area under the concentration–time curve; CI, confidence interval; C_max_, maximum plasma concentration; CV, coefficient of variation; ITZ, itraconazole; LRB, lurbinectedin; MPR, metabolite/parent ratio; n, number of patients with PK parameter included; NA, not applicable; PK, pharmacokinetic; R, Reference; RT, Reference–Test (ITZ + LRB in Cycle 2); T, Test; TR, Test–Reference (ITZ + LRB in Cycle 1).

**Table 4:** Treatment-related adverse events (>10% of patients or grade ≥ 3) and laboratory abnormalities (regardless of relationship) by study part (regardless of sequence), worst grade per treatment.

	Part A						Part B					
	ITZ + LRB ^a^ (*n* = 3)			LRB Alone (*n* = 3)			ITZ + LRB ^b^ (*n* = 10)			LRB Alone (*n* = 11)		
NCI-CTCAE Grade	All	3	4	All	3	4	All	3	4	All	3	4
**Treatment-related AEs**												
Nausea	–	–	–	–	–	–	1 (10)	–	–	5 (45)	–	–
Vomiting	–	–	–	1 (33)	–	–	–	–	–	3 (27)	1 (9)	–
Constipation	–	–	–	1 (33)	–	–	–	–	–	–	–	–
Fatigue	1 (33)	–	–	–	–	–	–	–	–	2 (18)	–	–
Rhabdomyolysis	–	–	–	–	–	–	–	–	–	1 (9)	1 (9)	–
Acute kidney injury	–	–	–	–	–	–	–	–	–	1 (9)	1 (9)	–
**Hematological laboratory abnormalities (regardless of relationship)**												
Anemia	3 (100)	–	–	3 (100)	–	–	10 (100)	–	–	9 (82)	1 (9)	–
Leukopenia	1 (33)	–	–	3 (100)	1 (33)	1 (33)	4 (40)	–	–	8 (73)	5 (45)	1 (9)
Lymphopenia	3 (100)	–	–	3 (100)	1 (33)	.	9 (90)	1 (10)	–	9 (82)	2 (18)	1 (9)
Neutropenia	.	–	–	2 (67)	–	2 (67)	2 (20)	–	–	8 (73)	5 (45)	3 (27)
Thrombocytopenia	1 (33)	–	–	2 (67)	–	–	4 (40)	–	–	8 (73)	–	1 (9)
**Biochemical laboratory abnormalities (regardless of relationship)**												
ALT increased	–	–	–	1 (33)	1 (33)	–	2 (20)	–	–	9 (82)	–	–
CPK increased	–	–	–	–	–	–	–	–	–	1 (9)	–	1 (9)
GGT increased	–	–	–	1 (33)	–	–	3 (30)	1 (10)	–	4 (36)	1 (9)	–

Values are *n* (%) of patients. ^a^ In Part A, LRB was given at 0.8 mg/m^2^ when co-administered with ITZ. ^b^ Based on exposure and safety experience in Part A, the dose of LRB when co-administered with ITZ was increased to 0.9 mg/m^2^. in Part B. AEs, adverse events; ALT, alanine aminotransferase; CPK, creatine phosphokinase; GGT, gamma-glutamyltransferase; ITZ, itraconazole; LRB, lurbinectedin; NCI-CTCAE, National Cancer Institute—Common Terminology Criteria for Adverse Events.

## Data Availability

The original contributions presented in the study have been included in the article and Supplementary Material; further inquiries can be directly addressed to the corresponding author. Preliminary results of this trial were presented at the European Society for Medical Oncology (ESMO) 2023 Annual Congress. “Moreno I, Hernández T, Calvo E, Fudio S, Kahatt C, Martínez S, Iglesias JL, Octavio R, Pérez-Ramos L, Montilla L, Zeaiter A and Lubomirov R. Lurbinectedin (LRB) pharmacokinetics (PK) and safety when co-administered with itraconazole (ITZ) in patients with advanced solid tumor. Abstract #6344”.

## Supplementary Materials

The following supporting information can be downloaded at: https://www.mdpi.com/article/10.3390/md22040178/s1, Table S1: Demographics and baseline characteristics of patients. Table S2: Treatment-emergent adverse events (regardless of relationship) by study part, worst grade per treatment.

## References

[1] Cuevas C., Francesch A. (2009). Development of Yondelis (Trabectedin, ET-743). A Semisynthetic Process Solves the Supply Problem. Nat. Prod. Rep..

[2] Cristina Mendonça Nogueira T., Vinicius Nora de Souza M. (2021). New Fda Oncology Small Molecule Drugs Approvals in 2020: Mechanism of Action and Clinical Applications. Bioorganic Med. Chem..

[3] Kim A.N., Ngamnithiporn A., Du E., Stoltz B.M. (2023). Recent Advances in the Total Synthesis of the Tetrahydroisoquinoline Alkaloids (2002–2020). Chem. Rev..

[4] Bueren-Calabuig J.A., Giraudon C., Galmarini C.M., Egly J.M., Gago F. (2011). Temperature-Induced Melting of Double-Stranded DNA in the Absence and Presence of Covalently Bonded Antitumour Drugs: Insight from Molecular Dynamics Simulations. Nucleic Acids Res..

[5] Jimeno A., Sharma M.R., Szyldergemajn S., Gore L., Geary D., Diamond J.R., Fernandez Teruel C., Soto Matos-Pita A., Iglesias J.L., Cullell-Young M. (2017). Phase I Study of Lurbinectedin, a Synthetic Tetrahydroisoquinoline That Inhibits Activated Transcription, Induces DNA Single- and Double-Strand Breaks, on a Weekly × 2 Every-3-Week Schedule. Investig. New Drugs.

[6] Costanzo F., Martínez Diez M., Santamaría Nuñez G., Díaz-Hernandéz J.I., Genes Robles C.M., Díez Pérez J., Compe E., Ricci R., Li T.K., Coin F. (2022). Promoters of Ascl1- and Neurod1-Dependent Genes Are Specific targets of Lurbinectedin in Sclc Cells. EMBO Mol. Med..

[7] Santamaría Nuñez G., Robles C.M., Giraudon C., Martínez-Leal J.F., Compe E., Coin F., Aviles P., Galmarini C.M., Egly J.M. (2016). Lurbinectedin Specifically Triggers the Degradation of Phosphorylated RNA Polymerase II and the Formation of DNA Breaks in Cancer Cells. Mol. Cancer Ther..

[8] Leal J.F., Martinez-Diez M., Garcia-Hernandez V., Moneo V., Domingo A., Bueren-Calabuig J.A., Negri A., Gago F., Guillen-Navarro M.J., Aviles P. (2010). PM01183, a new DNA Minor Groove Covalent Binder with Potent In Vitro and In Vivo Anti-tumour Activity. Br. J. Pharmacol..

[9] Singh S., Jaigirdar A.A., Mulkey F., Cheng J., Hamed S.S., Li Y., Liu J., Zhao H., Goheer A., Helms W.S. (2021). FDA Approval Summary: Lurbinectedin for the Treatment of Metastatic Small Cell Lung Cancer. Clin. Cancer Res..

[10] Markham A. (2020). Lurbinectedin: First Approval. Drugs.

[11] Trigo J., Subbiah V., Besse B., Moreno V., Lopez R., Sala M.A., Peters S., Ponce S., Fernandez C., Alfaro V. (2020). Lurbinectedin As Second-Line Treatment for Patients with Small-Cell Lung Cancer: A Single-Arm, Open-Label, Phase 2 Basket Trial. Lancet Oncol..

[12] Subbiah V., Brana I., Longhi A., Boni V., Delord J.P., Awada A., Boudou-Rouquette P., Sarantopoulos J., Shapiro G.I., Elias A. (2022). Antitumor Activity of Lurbinectedin, a Selective Inhibitor of Oncogene Transcription, in Patients with Relapsed Ewing Sarcoma: Results of a Basket Phase II Study. Clin. Cancer Res..

[13] Kristeleit R., Leary A., Delord J.P., Moreno V., Oaknin A., Castellano D., Shappiro G.I., Fernandez C., Kahatt C., Alfaro V. (2023). Lurbinectedin in Patients with Pretreated Endometrial Cancer: Results from a Phase 2 Basket Clinical Trial and Exploratory Translational Study. Investig. New Drugs.

[14] Longo-Munoz F., Castellano D., Alexandre J., Chawla S.P., Fernandez C., Kahatt C., Alfaro V., Siguero M., Zeaiter A., Moreno V. (2022). Lurbinectedin in Patients with Pretreated Neuroendocrine Tumours: Results from a Phase II Basket Study. Eur. J. Cancer.

[15] Boni V., Pistilli B., Brana I., Shapiro G.I., Trigo J., Moreno V., Castellano D., Fernandez C., Kahatt C., Alfaro V. (2022). Lurbinectedin, a Selective Inhibitor of Oncogenic Transcription, in Patients with Pretreated Germline BRCA1/2 Metastatic Breast Cancer: Results from a Phase II Basket Study. ESMO Open.

[16] Ganti A.K.P., Loo B.W., Bassetti M., Blakely C., Chiang A., D’Amico T.A., D’Avella C., Dowlati A., Downey R.J., Edelman M. (2021). Small Cell Lung Cancer, Version 2.2022, NCCN Clinical Practice Guidelines in Oncology. J. Natl. Compr. Cancer Netw..

[17] Dingemans A.C., Fruh M., Ardizzoni A., Besse B., Faivre-Finn C., Hendriks L.E., Lantuejoul S., Peters S., Reguart N., Rudin C.M. (2021). Small-Cell Lung Cancer: ESMO Clinical Practice Guidelines for Diagnosis, Treatment and Follow-Up. Ann. Oncol..

[18] Besse B., Paz-Ares L.G., Peters S., Cappuzzo F., Reck M., Calles A., Califano R., Lopez-Vilarino J.A., Veramendi S., Kahatt C.M. (2023). A Phase III Study of Lurbinectedin alone or in Combination with Irinotecan vs. Investigator’s Choice (Topotecan or Irinotecan) in Patients with Relapsed Small Cell Lung Cancer (SCLC; LAGOON Trial). J. Clin. Oncol..

[19] Fernandez-Teruel C., Gonzalez I., Trocóniz I.F., Lubomirov R., Soto A., Fudio S. (2019). Population-Pharmacokinetic and Covariate Analysis of Lurbinectedin (PM01183), a New RNA Polymerase II Inhibitor, in Pooled Phase I/II Trials in Patients with Cancer. Clin. Pharmacokinet..

[20] Liu L., Bello A., Dresser M.J., Heald D., Komjathy S.F., O’Mara E., Rogge M., Stoch S.A., Robertson S.M. (2016). Best Practices for the Use of Itraconazole as a Replacement for Ketoconazole in Drug-Drug Interaction Studies. J. Clin. Pharmacol..

[21] Chen Y., Cabalu T.D., Callegari E., Einolf H., Liu L., Parrott N., Peters S.A., Schuck E., Sharma P., Tracey H. (2019). Recommendations for the Design of Clinical Drug-Drug Interaction Studies With Itraconazole Using a Mechanistic Physiologically-Based Pharmacokinetic Model. CPT Pharmacomet. Syst. Pharmacol..

[22] Page R.L., O’Bryant C.L., Cheng D., Dow T.J., Ky B., Stein C.M., Spencer A.P., Trupp R.J., Lindenfeld J. (2016). Drugs That May Cause or Exacerbate Heart Failure: A Scientific Statement from the American Heart Association. Circulation.

[23] Machiels J.P., Staddon A., Herremans C., Keung C., Bernard A., Phelps C., Khokhar N.Z., Knoblauch R., Parekh T.V., Dirix L. (2014). Impact of Cytochrome P450 3A4 Inducer and Inhibitor on the Pharmacokinetics of Trabectedin in Patients with Advanced Malignancies: Open-Label, Multicenter Studies. Cancer Chemother. Pharmacol..

[24] Elez M.E., Tabernero J., Geary D., Macarulla T., Kang S.P., Kahatt C., Pita A.S., Teruel C.F., Siguero M., Cullell-Young M. (2014). First-in-Human Phase I Study of Lurbinectedin (PM01183) in Patients with Advanced Solid Tumors. Clin. Cancer Res..

[25] Lang I., Liu D., Fritsch H., Taube T., Chizhikov E., Liptai B. (2020). Potential Drug-Drug Interactions with Combination Volasertib + Itraconazole: A Phase I, Fixed-Sequence Study in Patients with Solid Tumors. Clin. Ther..

[26] Mu S., Lin C., Skrzypczyk-Ostaszewicz A., Bulat I., Maglakelidze M., Skarbova V., Andreu-Vieyra C., Sahasranaman S. (2021). The Pharmacokinetics of Pamiparib in the Presence of a Strong CYP3A Inhibitor (itraconazole) and Strong CYP3A Inducer (rifampin) in Patients with Solid Tumors: An Open-Label, Parallel-Group Phase 1 Study. Cancer Chemother. Pharmacol..

[27] Takahashi S., Karayama M., Takahashi M., Watanabe J., Minami H., Yamamoto N., Kinoshita I., Lin C.C., Im Y.H., Achiwa I. (2021). Pharmacokinetics, Safety, and Efficacy of Trastuzumab Deruxtecan with Concomitant Ritonavir or Itraconazole in Patients with HER2-Expressing Advanced Solid Tumors. Clin. Cancer Res..

[28] Faessel H., Nemunaitis J., Bauer T.M., Lockhart A.C., Faller D.V., Sedarati F., Zhou X., Venkatakrishnan K., Harvey R.D. (2019). Effect of CYP3A Inhibitors on the Pharmacokinetics of Pevonedistat in Patients with Advanced Solid Tumours. Br. J. Clin. Pharmacol..

[29] ZEPZELCA® (lurbinectedin) (2020). Prescribing Information.

[30] Leary A., Oaknin A., Trigo J.M., Moreno V., Delord J.P., Boni V., Brana I., Fernandez C., Kahatt C., Nieto A. (2023). Pooled Safety Analysis of Single-Agent Lurbinectedin in Patients with Advanced Solid Tumours. Eur. J. Cancer.

[31] Fernandez Teruel C., Lubomirov R., Fudio S. (2021). P48.23 Exposure-Response Analyses and Clinical Utility Index to Justify the Dosage of Lurbinectedin in Small-cell Lung Cancer. J. Thorac. Oncol..

[32] Gaillard S., Oaknin A., Ray-Coquard I., Vergote I., Scambia G., Colombo N., Fernandez C., Alfaro V., Kahatt C., Nieto A. (2021). Lurbinectedin Versus Pegylated Liposomal Doxorubicin or Topotecan in Patients with Platinum-Resistant Ovarian Cancer: A Multicenter, Randomized, Controlled, Open-Label Phase 3 Study (CORAIL). Gynecol. Oncol..

[33] King N., Garcia-Martinez S., Alcaraz E., Grisalena A., Lubomirov R., Altares R., Fernandez-Teruel C., Francesch A.M., Aviles P.M., Fudio S. (2023). Quantitative Determination of Lurbinectedin, Its Unbound Fraction and Its Metabolites in Human Plasma Utilizing Ultra-Performance LC-MS/MS. PLoS ONE.

