# Supplementary material for: Pharmacokinetics and Safety of Lurbinectedin Administrated with Itraconazole in Cancer Patients: A Drug–Drug Interaction Study
Source: Mar Drugs. 2024 Apr 16;22(4):178. doi: 10.3390/md22040178 (PMC11050816; doi:10.3390/md22040178)
Supplement: Supplementary file 1 [file marinedrugs-22-00178-s001.zip › marinedrugs-2941781-supplementary.pdf]

SUPPLEMENTARY MATERIAL

Table S1. Demographics and baseline characteristics of patients.

	**Part A**	**Part B**		**Total**
	**Seq. 1 (TR: ITZ + LRB Cycle 1) (n = 3)**	**Seq. 1 (TR: ITZ+LRB Cycle 1) (n = 5)**	**Seq. 2 (RT: ITZ+LRB Cycle 2) (n = 6)**	**(N = 14)**
**Gender**				
Male	–	3 (60%)	2 (33%)	5 (36%)
Female	3 (100%)	2 (40%)	4 (67%)	9 (64%)
**Median age, years (range)**	69 (49–72)	64 (56–70)	62 (56–72)	63 (49–72)
**ECOG performance status**				
0	1 (33%)	4 (80%)	5 (83%)	10 (71%)
1	2 (67%)	1 (20%)	1 (17%)	4 (29%)
**Median BSA, m^2 (range)**	1.60 (1.53–1.86)	1.70 (1.45–2.15)	1.76 (1.60–2.12)	1.73 (1.45–2.15)
**Stage at diagnosis**				
Early	1 (33%)	1 (20%)	2 (33%)	4 (29%)
Locally advanced	–	1 (20%)	2 (33%)	3 (21%)
Metastatic	2 (67%)	3 (60%)	2 (33%)	7 (50%)
**Primary tumors**				
Ovarian carcinoma	1 (33%)	–	3 (50%)	4 (29%)
Lung	–	1 (20%)	2 (33%)	3 (21%) [a]
Endometrial carcinoma	–	2 (40%)	–	2 (14%)
Colon adenocarcinoma	–	1 (20%)	–	1 (7%)
Epidermoid carcinoma	–	1 (20%)	–	1 (7%)
Leiomyosarcoma	–	–	1 (17%)	1 (7%)
Mesothelioma	1 (33%)	–	–	1 (7%)
Pancreatobiliary adenocarcinoma	1 (33%)	–	–	1 (7%)
**Number of sites**				
Median (range)	4 (2–4)	3 (2–5)	2 (1–5)	3 (1–5)
**Sites of disease**				
Lung	2 (67%)	4 (80%)	3 (50%)	9 (64%)
Lymph node	1 (33%)	4 (80%)	4 (67%)	9 (64%)
Liver	1 (33%)	2 (40%)	1 (17%)	4 (29%)
Bone	1 (33%)	1 (20%)	1 (17%)	3 (21%)
Peritoneum	1 (33%)	1 (20%)	1 (17%)	3 (21%)
Pleura	2 (67%)	1 (20%)	–	3 (21%)
Adrenal	–	1 (20%)	1 (17%)	2 (14%)
CNS	–	2 (40%)	–	2 (14%)
Breast	–	–	1 (17%)	1 (7%)
Kidney	–	–	1 (17%)	1 (7%)
Mesenteric lymph node	1 (33%)	–	–	1 (7%)
Muscle	–	–	1 (17%)	1 (7%)
Rib	1 (33%)	–	–	1 (7%)
**Time from diagnosis to first infusion (years)**				
Median (range)	2.6 (1.0–3.4)	3.4 (2.0–13.8)	3.5 (2.5–6.8)	3.4 (1.0–13.8)
**Prior treatment for advanced disease (prior chemotherapy lines)**				
Median (range)	2 (2–4)	3 (2–6)	5 (3–11)	4 (2–11)

Data shown are n (%) of treated patients by study part (Part A: Sequence 1 *versus* Part B: Sequence 1 and Sequence 2) and sequence (Sequence 1 [TR] *versus* Sequence 2 [RT]), except for median (range).

Part A/Sequence 1: ITZ 200 mg, once-daily for 12 days + LRB (0.8 mg/m^2, 1 h, IV infusion) (C1) followed by C2 and C3 of LRB alone; Part B/Sequence 1: ITZ 200 mg, once-daily for 12 days + LRB (0.9 mg/m^2, 1 h, IV Infusion) (C1) followed by C2 and C3 of LRB alone; Part B/Sequence 2: LRB alone (3.2 mg/m^2, 1 h, IV infusion) (C1) followed by ITZ 200 mg, once-daily for 12 days + LRB (0.9 mg/m^2, 1 h, IV infusion) (C2) and LRB alone (C3). LRB was administered at 3.2 mg/m^2 as a 1 h IV infusion q3wk for all patients when given without ITZ.

[a] Lung (n = 3 patients) included: NSCLC (n = 2 patients) and SCLC (n = 1 patient).

BSA, body surface area; CNS, central nervous system; ECOG, Eastern Cooperative Oncology Group; ITZ, itraconazole; LRB, lurbinectedin; NSCLC, non-small cell lung cancer; PS, performance status; q3wk, every three weeks; RT, Reference–Test (ITZ + LRB in Cycle 2); Seq.1, Sequence 1; Seq. 2, Sequence 2; SCLC, small cell lung cancer; TR, Test–Reference (ITZ + LRB in Cycle 1).

Table S2. Treatment-emergent adverse events (regardless of relationship) by study part, worst grade per treatment.

	Part A						Part B					
	ITZ+LRB (n = 3)			LRB alone (n = 3)			ITZ+LRB (n = 10)			LRB alone (n = 11)		
**NCI-CTCAE grade**	**All Grades**	**3**	**4**	**All Grades**	**3**	**4**	**All Grades**	**3**	**4**	**All Grades**	**3**	**4**
**Gastrointestinal disorder**												
Nausea	—	—	—	—	—	—	1 (10)	—	—	5 (45)	—	—
Vomiting	—	—	—	1 (33)	—	—	—	—	—	3 (27)	1 (9)	—
Diarrhea	—	—	—	-	—	—	—	—	—	1 (9)	—	—
Constipation	—	—	—	1 (33)	—	—	—	—	—	—	—	—
Abdominal pain	—	—	—	—	—	—	1 (10)	—	—	—	—	—
Intestinal obstruction	—	—	—	—	—	—	1 (10)	1 (10)	-	—	—	—
Intestinal pseudo-obstruction	—	—	—	—	—	—	—	—	—	1 (9)	—	—
Small intestinal obstruction	—	—	—	—	—	—	—	—	—	1 (9)	1 (9)	—
Stomatitis	—	—	—	—	—	—	—	—	—	1 (9)	—	—
**General disorders and administration site conditions**												
Fatigue	1 (33)	—	—	1 (33)	—	—	—	—	—	2 (18)	—	—
**Infections and infestations**												
Stoma site infection	—	—	—	—	—	—	1 (10)	—	—	—	—	—
**Respiratory, thoracic and mediastinal disorders**												
Respiratory failure	—	—	—	—	—	—	—	—	—	1 (9)	—	—
**Metabolism and nutrition disorders**												
Decreased appetite	—	—	—	—	—	—	1 (10)	—	—	—	—	—
Hypercalcemia	—	—	—	—	—	—	1 (10)	—	—	—	—	—
**Musculoskeletal and connective tissue disorders**												
Bone pain	1 (33)	—	—	1 (33)	—	—	—	—	—	—	—	—
Back pain	—	—	—	—	—	—	1 (10)	—	—	—	—	—
Rhabdomyolysis	—	—	—	—	—	—	—	—	—	1 (9)	1 (9)	—
**Nervous system disorders**												
Syncope	1 (33)	1 (33)	—	—	—	—	—	—	—	—	—	—
Dysgeusia	—	—	—	1 (33)	—	—	—	—	—	—	—	—
**Neoplasms benign, malignant and unspecified (including cysts and polyps)**												
Tumor pain	—	—	—	—	—	—	1 (10)	—	—	—	—	—
**Renal and urinary disorders**												
Acute kidney injury	—	—	—	—	—	—	—	—	—	1 (9)	1 (9)	—
**Respiratory, thoracic and mediastinal disorders**												
Cough	1 (33)	—	—	—	—	—	—	—	—	—	—	—
Dyspnea	1 (33)	—	—	—	—	—	—	—	—	—	—	—
Pleural effusion	1 (33)	—	—	1 (33)	1 (33)	—	—	—	—	—	—	—
**Vascular disorders**												
Deep vein thrombosis	—	—	—	—	—	—	—	—	—	1 (9)	1 (9)	—

Values are n (%) of patients.
ITZ, itraconazole; LRB, lurbinectedin; NCI-CTCAE, National Cancer Institute Common Terminology Criteria for Adverse Events.
